# The metastasis-associated protein S100A4 exists in several charged variants suggesting the presence of posttranslational modifications

**DOI:** 10.1186/1471-2407-8-172

**Published:** 2008-06-13

**Authors:** Mads H Haugen, Kjersti Flatmark, Svein-Ole Mikalsen, Gunhild M Malandsmo

**Affiliations:** 1 Dept. of Tumor Biology, Institute for Cancer Research, Norwegian Radium Hospital, Rikshospitalet University Hospital, 0310 Oslo, Norway; 2 Dept. of Surgical Oncology, Norwegian Radium Hospital, Rikshospitalet University Hospital, 0310 Oslo, Norway; 3 Norwegian Radium Hospital Faculty Division, University of Oslo, 0310 Oslo, Norway; 4 Dept. of Cancer Prevention, Institute for Cancer Research, Norwegian Radium Hospital, Rikshospitalet University Hospital, 0310 Oslo, Norway

## Abstract

**Background:**

S100A4 is a metastasis-associated protein which has been linked to multiple cellular events, and has been identified extracellularly, in the cytoplasm and in the nucleus of tumor cells; however, the biological implications of subcellular location are unknown. Associations between a variety of posttranslational protein modifications and altered biological functions of proteins are becoming increasingly evident. Identification and characterization of posttranslationally modified S100A4 variants could thus contribute to elucidating the mechanisms for the many cellular functions that have been reported for this protein, and might eventually lead to the identification of novel drugable targets.

**Methods:**

S100A4 was immuoprecipitated from a panel of *in vitro *and *in vivo *sources using a monoclonal antibody and the samples were separated by 2D-PAGE. Gels were analyzed by western blot and silver staining, and subsequently, several of the observed spots were identified as S100A4 by the use of MALDI-TOF and MALDI-TOF/TOF.

**Results:**

A characteristic pattern of spots was observed when S100A4 was separated by 2D-PAGE suggesting the presence of at least three charge variants. These charge variants were verified as S100A4 both by western immunoblotting and mass spectrometry, and almost identical patterns were observed in samples from different tissues and subcellular compartments. Interestingly, recombinant S100A4 displayed a similar pattern on 2D-PAGE, but with different quantitative distribution between the observed spots.

**Conclusion:**

Endogenously expressed S100A4 were shown to exist in several charge variants, which indicates the presence of posttranslational modifications altering the net charge of the protein. The different variants were present in all subcellular compartments and tissues/cell lines examined, suggesting that the described charge variants is a universal phenomenon, and cannot explain the localization of S100A4 in different subcellular compartments. However, the identity of the specific posttranslational modification and its potential contribution to the many reported biological events induced by S100A4, are subject to further studies.

## Background

S100A4 is a small (approximately 12 kDa) acidic calcium-binding protein that has been associated with a range of biological functions, such as cell migration, invasion and angiogenesis, potentially contributing to higher metastatic capacity of tumor cells [1-4]. In line with this, increased expression of S100A4 has been correlated with adverse prognosis in patients with various types of cancer [5].

S100A4 belongs to the S100 protein family comprising at least 20 members, of which all are exclusively expressed in vertebrates. The human variant of S100A4 consists of 101 amino acids and is characterized by two calcium-binding EF-hands connected by an intermediate region referred to as the hinge region, and a distinct C-terminal extension. The S100 protein family shows a high degree of sequence homology, especially in the calcium binding EF-hand regions, while the composition of the C-terminal extension and the hinge region is more diversified and thus characterize each member [6,7]. Numerous studies indicate that S100A4 is arranged as homodimers held together by non-covalent bonds, and that this dimerization is important for the biological function. Upon calcium binding the homodimer undergo a conformational change that leads to exposure of the hydrophobic regions in the C-terminal end, initially buried in the complex [8].

S100A4 is located both in the cytoplasm, extracellularly [9], and also in the nucleus of tumor cells [10], but the mechanisms for transport and homing to subcellular compartments remains largely unexplored. The protein has also been found expressed in a variety of different normal cells [11]and the release of S100A4 into the extracellular space may thus originate both from tumor and/or stromal cells [12].

Most of the reported intracellular effects of S100A4 are associated with cytoskeleton rearrangements that may influence cellular motility [13-17], and extracellulary added S100A4 has also been shown to boost migration of astrocytic tumor cells [18]. In addition, the extracellulary added protein may sensitize osteosarcoma cells to INF-γ mediated apoptosis [19], and provoke degradation of the extracellular matrix (ECM) by augmenting the levels of matrix metalloproteinases [20]. The fact that S100A4 induces remodeling of the ECM suggests that the protein may also affect angiogenesis [21]. The mechanism by which S100A4 exerts its many reported and partly contradictory biological functions is not well understood, but one hypothesis could be that the protein exhibits different functions depending on its subcellular localization and/or posttranslational modifications.

A vast number of posttranslational protein modifications (PTMs) have been described [22]. PTMs may result in physiochemical changes of the protein with respect to mass, charge, structure and conformation, and thereby alter functional properties of the protein, such as binding affinity, enzyme activity and protein hydrophobicity. As a consequence, PTMs may target a protein for compartmentalization, degradation and protein-protein interactions. During the last few years progression in genomic and proteomic technology has pushed the limits of protein knowledge and one has realized the functional importance of several novel PTMs other than the more common. As an example, human p53 has been reported to be potentially post-translationally modified on at least 18 sites [23] including phosphorylation, acetylation and sumoylation, with implications for DNA binding, stability, oligomerization, nuclear import/export and ubiquitination [24].

The localization of S100A4 in the different cellular compartments suggests some type of subcellular targeting and transport. The present study was undertaken to investigate whether PTM of S100A4 could cause specific localization of the protein. Such modifications could, through increased calcium binding potential or altering the functional C-terminal extension, also have impact on the biological function of the protein when localized in the particular compartments. To study this we isolated S100A4 from several tissues, cellular compartments and also from recombinant sources and analyzed the protein by 2D-PAGE and western blotting. The S100A4 identity of several observed 2D spots were subsequently confirmed by the use of mass spectrometry (MALDI-TOF).

## Methods

### Cell lines and cell culture

Human colorectal carcinoma cell lines HCT116 and SW620 were purchased from ATCC (Manassas, VA) and the OHS osteosarcoma cell line was established in-house [25]. All cell lines were cultured in RPMI-1640 (Bio Whittaker, Verviers, Belgium) containing 10% fetal bovine serum (PAA Laboratories GmbH, Pasching, Austria), 20 mM Hepes and 2 mM glutamax (GIBCO BRL Life Technologies, Paisley, UK). Cell cultures were routinely tested negative for mycoplasma infection. Cells were detached using EDTA (Bio Whittaker) and washed twice in PBS (Bio Whittaker).

### Patient samples

Tumor tissue was obtained from colorectal cancer patients during primary surgery, snap-frozen in liquid nitrogen and stored at -80°C. Eight tumor biopsies were selected from a panel of primary colorectal carcinoma samples, including two tumors from each Duke's stage (A, B, C and D). All tumors expressed high levels of S100A4, as assessed previously by immunohistochemistry, and within each stage group, one tumor harboring wt and one mutated *TP53 *were selected [10].

### Isolation of red blood cells (RBC) and mononuclear cells (MNC)

Human red blood cells and mononuclear cells were isolated from whole blood samples from healthy donors. Red blood cells were isolated by gradient centrifugation using CPT tubes (Becton Dickinson Co., Franklin Lakes, NJ) according to the manufacturers' instruction, and the fraction below the gel was collected. Mononuclear cells were isolated by Lymphoprep density centrifugation (Medinor, Oslo, Norway) according to the manufacturers' instructions and collected from the interphase layer. Protein isolation was performed as described below.

### Recombinant S100A4

One of the human recombinant S100A4 variants was from Jena Biosciences (Jena, Germany), the second was a kind gift from Prof. E. Lukanidin (Danish Cancer Society, Copenhagen, Denmark), and the third was produced in-house by the use of the prokaryot expression vector pGEX 3X (GE Lifesciences, Uppsala, Sweden) inserting the S100A4 sequence into the *BamHI *site and purified using Glutathione Sepharose 4B (GE Lifesciences). The GST-tag was cleaved by Factor Xa treatment and subsequent purification using the Factor Xa cleavage capture kit (Novagen, La Jolla CA), all according to the manufacturers protocol. The resulting recombinant S100A4 contains Glycine, Isoleucine and Proline C-terminally in addition to the native human sequence. Mouse recombinant S100A4 was produced using the *E. coli *vector pQE30 (Qiagen, Hilden, Germany) using the *BamHI *insertion site which resulted in C-terminal attachment of RGS-6xHis and Proline-Arginine to the native mouse sequence. The recombinant mouse S100A4 was purified using Talon Metal Affinity Resin (Clontech, Mountain View, CA), according to the manufacturers' protocol. All proteins were stored in PBS at -70°C.

### Preparation of protein lysates

Preparation of whole cell lysates was performed by addition of lysis buffer (150 mM NaCl, 50 mM Tris-HCl pH 7.5, 0.1% NP-40, 10 μg/ml of each leupeptin hemisulfate, aprotinin, pepstatin A, 20 mM β-glycerolphosphate, 1 mM PMSF, 1 mM sodium orthovanadate and 100 mM sodium fluoride) to dry cell pellets, left on ice for 1 h, sonicated and centrifuged to remove cell debris.

Extracellular protein fractions were prepared by collecting growth medium from confluent grown SW620 cells. The collected growth medium was analyzed for dead/lysed cells by measuring levels of the intracellular constituent lactate dehydrogenase (data not shown). Finally the conditioned growth medium was concentrated by reducing the volume to 1/10 in a rotavapor (Thermo Savant, Waltham, MA) and desalted using Zeba Desalt Spin Columns (Pierce, Rockford, IL).

Sub-fractioning of nuclear and cytoplasmic components was performed essentially as previously described [26] with some modifications. Cells were mixed with buffer B (340 mM trisodium citrate, 100 mM NaCl and 1% Triton), left on ice for 30 min. and homogenized in a Potter-Elvehjem homogenizer (10 strokes at 2000 rpm). The homogenate was mixed with cold STKM buffer (250 mM sucrose, 50 mM Tris-HCl pH 7.5, 25 mM KCl and 5 mM MgCl_2_) and the nuclear fraction isolated by centrifugation (37000 rpm for 30 min in a 55Ti rotor at 4°C). The supernatant (cytoplasmic fraction) was collected and nuclear lysates prepared as described above. Purity of the fractions was assessed by western blot, staining for α-tubulin as cytoplasmic marker (CP06, Calbiochem, San Diego, CA) and lamin B as nuclear marker (NA12, Calbiochem) as described in detail previously [10].

Frozen tissue samples (15–24 mg) were blended in lysis buffer (20 mM Tris pH 7.5, 137 mM NaCl, 100 mM sodium fluoride, 10% glycerol, 1% NP-40, 10 μg/ml leupeptin hemisulfate, aprotinin, pepstatin A, 20 mM β-glycerophosphate, 1 mM PMSF, 1 mM sodium orthovanadate) using a homogenizer (IKA Labortechnik, Staufen, Germany), left on ice for 1 h, sonicated and then centrifuged to remove cellular debris.

Protein concentration from all lysates, except from red blood cells due to hemoglobulin interference, was estimated using BCA Protein Assay Kit (Pierce) and lysates were stored at -80°C until use.

### Immunoprecipitation (IP)

An in-house monoclonal antibody MAb 20.1 [27] was coupled to CNBr activated Sepharose 4B (GE Lifesciences) according to the manufacturer's protocol and stored in 20% EtOH solution at 4°C. The protein lysate was mixed with 60 μl conjugated beads and IP buffer (300 mM NaCl, 0.25% Na-lauroyl sarcosin, 0.45% Na-deoxy cholate, 100 mM Na-phosphate buffer pH 8.2) and incubated with rotation at 4°C over night. After a short spin in a bench top centrifuge the supernatant was discarded and the beads washed twice in IP buffer and transferred to Spin-X Centrifuge Tube Filter 0.22 μm (Corning Incorporated, Corning, NY). The precipitate was eluted in H_2_O by heating at 95°C for 1 minute and spinning for 2 minutes at 4000 rpm, followed by desalting using Zeba Desalt Spin Columns (Pierce) and finally concentrated in a rotavapor (Thermo Savant, Waltham, MA).

### 2D-gel electrophoresis and staining

2D-gel electrophoresis was performed using the ZOOM IPG-Runner System (Invitrogen, Carlsbad CA, USA) with immobilized pH gradient (IPG) strips pH 4–7 and gradient SDS-PAGE 4–12% in 2-(N-morpholino)ethanesulfonic acid (MES) buffer. All procedures were performed according to the manufacturer's protocol, which includes reduction and alkylation of cysteine residues. De-ionizing of urea (Kodak, Rochester, NY), used in the sample rehydrating buffer, was performed where indicated by addition of mixed-bed resin (BioRad, Hercules, CA). The proteins were transferred to a 0.22 μm polyvinylidine difluoride membrane (Millipore Corporation, Bedford, MA) in a Mini Trans-Blot Cell (BioRad). Detection of S100A4 was performed as previously described using an in-house produced monoclonal antibody MAb 22.3 [27] or a commercially available polyclonal antibody (DAKO, Glostrup, Denmark). Silver staining and destaining were essentially performed as previously described [28]. When recombinant protein was run on 2D-PAGE, a small portion of antibody was mixed in the sample for alignment with previous results where S100A4 was isolated by immunoprecipitation.

### MALDI-TOF preparation and analysis

Spots of interest were excised from silver stained 2D-PAGE, destained and washed twice (25 mM NH_4_HCO_3_, 50% acetonitrile) before samples were subjected to in gel protein digestion using sequence grade modified trypsin (Promega, Madison, WI). Reduction and alkylation was performed during 2D-PAGE, and was consequently not repeated. Peptides were extracted in a mixture of Trifluoroacetic acid (TFA) and acetonitrile, concentrated and desalted using C_18 _affinity material (Empore Extraction disks, 3 M, St. Paul, MN) assembled in a pipette tip, mixed with α-cyano-4-hydroxycinnamic acid (Fluka/Sigma-Aldrich, St. Louis, MO) and spotted on a 384-well steel AnchorChip sample plate (Bruker Daltonics, Billerica, MA). Prior to desalting, some peptide samples were also treated using ProteoMass Guanidation Kit (Sigma-Aldrich, St. Louis, MO) according to the manufacturer's protocol. MS analysis was carried out in an Ultraflex^® ^MALDI-TOF/TOF instrument (Bruker Daltonics) and the obtained spectra analyzed in the FlexAnalysis software.

### Electro-elution from 2D-PAGE

20 μg of recombinant S100A4 were run on two identical 2D-PAGEs of which one was stained with coomassie-blue (Fermentas, Burlington, ON) to use as template for cutting out spots of interest from the untreated gel. Protein from the excised spots was eluted in ElutaTube (Fermentas) using running buffer (200 mM glycine, 2 mM Tris-HCl, 0.025% SDS) at 100 V for 60 minutes. The samples were then precipitated according to the manufacturer's protocol, air dried and re-suspended in H_2_O. Re-running of 2D-PAGE was performed as described above.

### Computerized prediction algorithms for phosphorylation and deamidation

The primary amino acid sequence of S100A4 in FASTA format (SwissProt entry P26447) was submitted (accessed Nov. 2006) to the NetPhos 2.0 Server [29], which returns prediction scores between 0 and 1 for possible phosphorylations on Serine, Threonine and Tyrosine residues. Similarly, the solution structure of S100A4 (PDB entry 1m31) [30] was submitted to the Deamidation CD and ID Search Form (accessed Nov. 2006) [31], which calculates a CD score for each Asparagine residue reporting their predicted half life in 0.15 M Tris-HCl buffer pH 7.4 at 37°.

## Results

### Characteristic distribution pattern of human S100A4 when separated by 2D-PAGE

**Figure 1:**
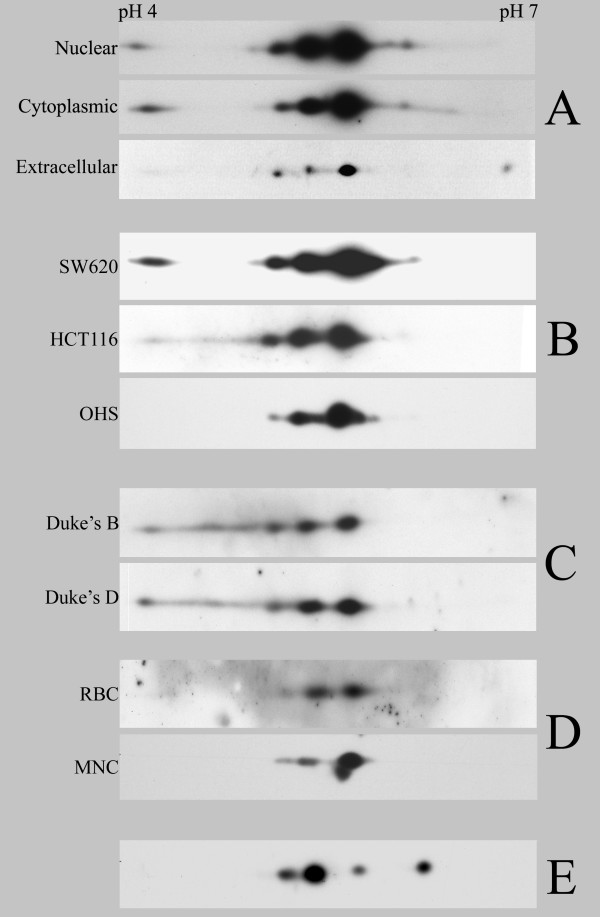
**Western blot of S100A4 separated by 2D-PAGE**. S100A4 was immunoprecipitated from the complex samples using MAb 20.1 and blotted onto PVDF filters. The western blots are visualized by staining with MAb 22.3 against S100A4 and aligned according to the antibody light chain (not shown) detached during the immunoprecipitation procedure or added directly into the recombinant samples. A. Comparison of S100A4 in nuclear and cytoplasmic fractions isolated from *in vitro *cultured colorectal cancer cell line SW620 and extracellular S100A4 from conditioned growth medium by confluently growing SW620 cells. B. Comparison of S100A4 isolated from whole cell lysate of two *in vitro *cultured colorectal cancer cell lines with different TP53 status, SW620 (*TP53 *mut) and HCT116 (*TP53 *wt), and the OHS osteosarcoma cell line. C. Analysis of S100A4 isolated from biopsies collected from colorectal cancer patients diagnosed with different p53 status and Duke's stage. Two representative samples out of eight analyzed are shown. D. Endogenous S100A4 isolated from red blood cells (RBC) and mononuclear cells (MNC). E. Recombinant human S100A4 produced in-house using vector pGEX 3X (with removal of GST-tag).

A characteristic pattern of spots was observed when S100A4 immunoprecipitated from SW620 cell lysate was separated by 2D-PAGE, blotted and stained with anti-S100A4 (Fig. 1). At least three, in some cases four, distinct spots were observed corresponding to the expected molecular mass of the protein. The position of the most intense spot was consistent with the theoretical mass and isoelectric point (pI) of human S100A4 as predicted by ProtParam on the Expasy web page (MW = 11728, pI = 5.85). A trail of two or three gradually less intense spots separated by approximately 0.2–0.3 pH units were observed towards the more acidic end, with the most acidic spot located at pH 4. Furthermore, when cytoplasmic and nuclear fractions isolated from *in vitro *cultured SW620 cells were compared with extracellular protein by similar analysis, almost identical distribution patterns were observed (Fig. 1A).

Previously, expression of S100A4 was suggested to depend upon p53 mutational status [32], and it was therefore of interest to examine whether this could be of importance for the observed distribution pattern on 2D-PAGE. By comparing immunoprecipitated S100A4 from HCT116 with the above results from SW620 no apparent differences between cell lines harboring wild-type and mutated *TP53*, respectively, were revealed. In addition, when examining S100A4 from the osteosarcoma cell line OHS, the same pattern was observed (Fig. 1B).

Although a consistent 2D-PAGE distribution pattern was observed in all the samples from the two cell lines, different results might be expected when analyzing patient material. *In vitro *cell culture lack the contribution from the normal microenvironment, and changes in the genetic profile may also be induced due to long term cultivation. When immunoprecipitated protein from a panel of eight primary colorectal carcinoma biopsies was examined by 2D-PAGE, no clear differences compared to the *in vitro *samples were detected, except for variations attributable to varying amounts of total S100A4. Moreover, no differences were observed according to tumor stage or *TP53 *mutational status as indicated by the representative samples shown in Fig. 1C.

### 2D patterns of S100A4 from normal cells and recombinant sources

**Figure 2:**
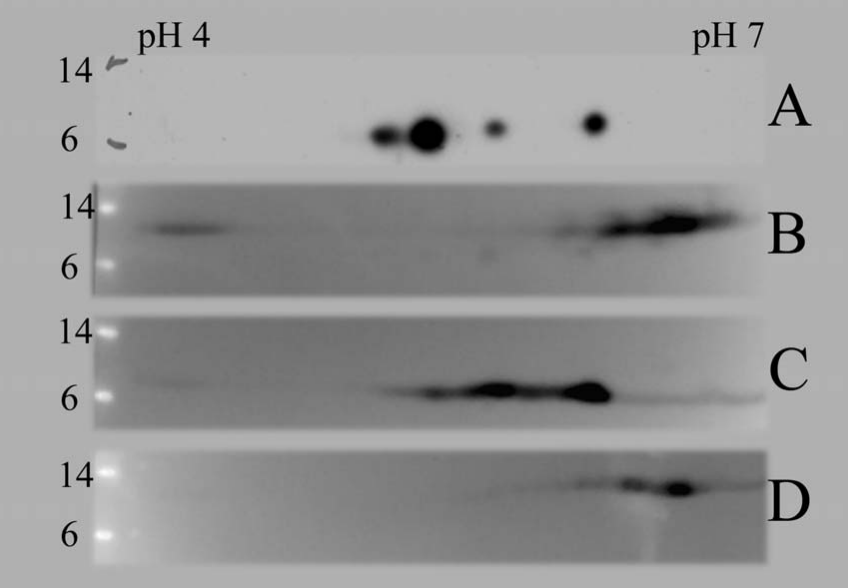
**2D-PAGE distribution pattern of a panel of recombinant (*E. coli*) S100A4 proteins**. One μg recombinant protein was separated by 2D-PAGE and visualized by western blots staining with a polyclonal antibody recognizing both mouse and human S100A4. A: Human S100A4 produced in-house using vector pGEX 3X with removal of GST-tag (previously shown in fig. 1E). B: Human his-tagged S100A4 produced in the lab of Prof. E. Lukanidin (Danish Cancer Society). C: Human S100A4 produced by Jena Biosciences. D: Mouse his-tagged S100A4 produced in-house using vector pQE30.

Having observed several presumably modified forms of S100A4 in cancer cells it was of interest to examine whether this was the case also in normal human cells. In previous studies, RBC [27] and MNC [33,34] were found to have high expression of the protein. When S100A4 was immunoprecipitated from these normal cells and analyzed by 2D-PAGE the same distribution as described for S100A4 isolated from cancer cells was observed (Fig. 1D). In contrast, when recombinant human S100A4 produced in *E. coli *was separated by 2D-PAGE and visualized as before, a rather similar 2D pattern to the one previously observed for human endogenously expressed protein was obtained, but with considerable differences in the quantitative distribution between the spots (Fig. 1E). Separation into more than one spot was also observed for other recombinant S100A4, encoding either the human or mouse variant, but all produced in *E. coli *(Fig. 2).

To exclude the possibility that the observed pattern could have resulted from changes introduced during sample processing, two control experiments was performed. First, in-house produced human recombinant S100A4 was subjected to 2D-PAGE, extracted from one intense spot, re-run on 2D-PAGE and stained with MAb 22.3. The result of this visualization was a single spot located at the same MW and pI as the extracted spot (data not shown). Furthermore, carbamylation of proteins by isocyanic acid, a degradation product of urea, could also increase net negative charge and thus result in the observed pattern. However, running two samples of endogenous S100A4 in parallel, one where all degradation products of urea was cleared away by using mixed bed resin prior to mixing with S100A4, and one with no clearing, resulted in identical patterns. These two experiments indicated that our handling procedures did not induce modifications in the protein sequence that could cause the observed pattern on the 2D-PAGE.

### Verification of 2D spots as S100A4

**Figure 3:**
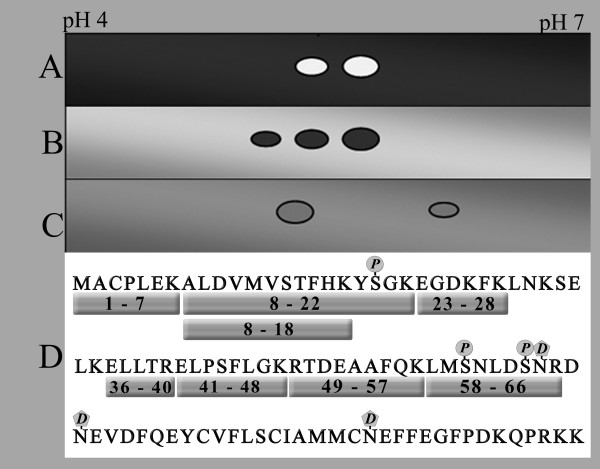
**Charge variants identified as S100A4 by MALDI-TOF, detected tryptic peptides, and location of predicted PTMs**. A-C: Schematic representation of the different spots obtained from various sources confirmed as S100A4 by MALDI-TOF and MALDI-TOF/TOF. All shown spots are identified in the SwissProt library as S100A4 with p < 0.05. A: *In vitro *cultured HCT116. B: Tumor biopsy from a colorectal cancer patient. C: Human recombinant S100A4 produced in-house. D: Representation of the S100A4 amino acid sequence with all tryptic peptides discovered on MALDI-TOF (boxes) and location of phosphorylations (P) and deamidations (D) predicted by computer algorithms.

Although the antibodies against S100A4 used for immunoprecipitation and western blot analyses (MAb 20.1 and MAb 22.3) both have been proven to be highly specific, the risk of false positive results because of cross reactivity cannot be excluded. Thus, to further confirm the identity of the detected S100A4 variants, regions of interest were excised from gels and in-gel tryptic digestion performed, upon which peptides were extracted and run on MALDI-TOF. When necessary for identification, MALDI-TOF/TOF was run on a peptide, representing amino acids 8–18 of the 101 in total for S100A4. When analyzing the produced mass list through Mascot [35] using peptide mass fingerprinting (PMF) and MS/MS fragment ion series towards the SwissProt library, a score corresponding to p < 0.05 for match in the library (SwissProt entry P26447) was considered as a positive identification. Using this strategy, two excised spots from SW620 whole cell lysate, three from a primary colorectal carcinoma biopsy and two from in-house produced recombinant S100A4 were confirmed to contain human S100A4. All these identified spots are indicated in a schematic presentation (Fig. 3A–C).

When pooling every detected tryptic peptides from all samples, these correspond to the entire list of theoretically predicted peptides of S100A4 in the range of 500–2000 Da (Fig. 3D). However the coverage of the S100A4 amino acid sequence was subject to variations in the range of 30–50% in the different experiments and the measured m/z of some tryptic peptides could be attributed to more than one theoretically predicted peptide sequence within S100A4.

The S100A4 amino acid sequence was analyzed with respect to two possible modifications using web based PTM prediction algorithms. The analysis returned three Serine residues, S20, S60 and S64 with scores of 0.997, 0.846 and 0.996, respectively, suggesting that these residues have high probability of being phosphorylated. Furthermore, analysis of the solution structure of S100A4 revealed three potentially unstable Asparagine residues N65, N68 and N87 with CD values of 8.293, 21.696 and 13.500, respectively, which suggests that these residues could be subject to deamidation. The S100A4 amino acid chain and location of predicted modifications are schematically illustrated (Fig. 3D).

## Discussion

In the present work we have demonstrated that endogenous human S100A4 distributes in a characteristic pattern when separated by 2D-PAGE, and irrespective of source or subcellular compartment used for immunoisolation of the protein, the observed pattern was almost identical. Interestingly, a similar pattern, but with different quantitative distribution, was observed when analyzing different batches of recombinant S100A4. The identity of several examined spots was confirmed for the first time by mass spectrometric methods, verifying the existence of at least three charge variants of S100A4. The most likely interpretation of the presence of these different charge variants is that S100A4 is posttranslationally modified, since changes introduced during sample processing was largely excluded.

Immunoprecipitated human S100A4 was separated by 2D-PAGE in at least three (in some cases four) distinct spots of equivalent mass but with different charge. A comparable observation, although verified only by immunological methods, was made when S100A4 from tumor interstitial fluid from breast cancer patients was separated by 2D-PAGE [11]. Furthermore, in a recently published study on p53-related changes, two different PTMs of S100A4 were indicated by SELDI-TOF-MS [36]. These modifications, glutathionylation and cysteinylation, could theoretically have caused the distribution pattern observed in this study, however preliminary experiments indicate that this is not the case.

The finding that human S100A4 isolated from primary colorectal carcinomas, cell lines and normal cells resulted in similar patterns is remarkable and suggests that the observed 2D-PAGE distribution is a general phenomenon. This was also underscored by the identification of an almost identical pattern when the protein was isolated from nuclear, cytoplasmic and extracellular compartments. This is unanticipated since PTM often is transiently present and the occurrence is frequently time-, location- and site specific [37].

Surprisingly, all four recombinant S100A4 proteins exhibited more than one charge variant when separated by 2D-PAGE. Because of its relative ease in manipulation, *E. coli *has emerged as the most common hosts for heterologous protein production, however, one major disadvantage hamper its use; the inability to introduce important PTMs such as glycosylation, phosphorylation and acylation, which may be essential for biological activity [38]. In contrast to the pattern observed for endogenous S100A4, none of the recombinant proteins clearly displayed the characteristic picture of one main spot followed by gradually less intense spots, which would be expected for proteins altered by sequential PTMs. The choice of cloning strategy may result in the addition of extra amino acids and affinity tags on recombinant proteins that could influence 2D-PAGE migration patterns, potentially complicating the interpretation of such data; however, one might expect that the final protein distributed in one single spot. The observed variable distribution could possibly be linked to differences in biological effect when using different recombinant proteins. In theory, the observed distribution pattern of the recombinant variants could be caused by heterogeneous cleavage of the selection tags (GST/His) or by unspecific degradation, but examination of undigested samples by MALDI-MS did not support such an explanation (data not shown).

Since immunological methods depend on antibody specificity and the S100 protein family exhibits high sequence homology, the confirmation of the present results by non-immunological methods was warranted. Using MALDI-TOF mass spectrometry to analyze tryptic peptides from excised gel spots, the identity of several spots reproducibly confirmed the results obtained by western blotting. Unfortunately, we have so far not been able to identify and further characterize the present PTMs. One possible explanation could be that the modified fragments were undetectable since the sequence coverage of this small protein was only between 30 and 50%. Also, trypsin digestion might cleave potentially interesting parts of the molecule into fragments that are too small for further analysis. We are in the process of establishing protocols using other digestive enzymes and MALDI-matrixes ensuring better coverage of the protein sequence.

Although the actual PTMs have not been identified, the observed 2D pattern gives several indications with respect to the type of the putative modifications. Since the apparent mass did not change significantly between the spots, all PTMs introducing heavy residues or major deletions in the amino acid sequence can be excluded. Furthermore, the visual impression of the 2D pattern suggests the presence of one quantitatively dominating variant, with less abundant variants distributing towards lower pH, which could indicate either successive loss of positive charges or gain of negative charges, resulting in gradually more acidic forms of the protein. Even with these limitations a large number of PTMs are possible from a physicochemical point of view, and although a comprehensive analysis cannot be made, it is tempting to speculate on a few of the more likely alternatives. Phosphorylation introduces a negative charge on Serine, Threonine or Tyrosine residues while only changing the mass by 80 Da. Analyzing the S100A4 amino acid sequence in NetPhos revealed three Serine residues as likely phosphorylation sites, and the observed 2D pattern match well with the predicted pattern if such modifications were present. Another possible modification that would introduce a negative charge with minimal mass change is deamidation, in which Asparagine or Glutamine is converted to Aspartic or Glutamic acid. A prediction algorithm for deamidation of S100A4 suggested the presence of three labile Asparagines which, if sequentially deamidated, theoretically would produce a 2D pattern similar to the one observed in our experiments. Another frequently occurring PTM involves cleavage of peptide residues resulting in loss of amino acids either from the N- or C-terminal end. Such loss of the three most C-terminal amino acids in S100A4 might also explain the observed pattern. All three PTMs suggested above could potentially have an impact on biological function, subcellular targeting or protein interaction of S100A4.

It is becoming increasingly evident that PTMs may be involved in the modulation of protein synthesis, degradation, activity, and molecular interactions, of which all may have implications for multiple cellular events [39,40]. S100A4 has been associated with many biological functions, some of which are probably related to localization in specific tissues and cellular compartments, and it may be possible that PTMs could be a permissive requirement for the exertion of some of the functions attributed to this protein.

Given the high sequence homology within the S100 family, modifications observed in other S100-proteins might also be relevant for S100A4, even if individual family members have discrete functions and tissue distribution. The S100A11 protein has been found translocated to the nucleus upon phosphorylation [41], and we therefore hypothesized that phosphorylation could also be involved in translocating S100A4 to the nucleus. The observed comparable distribution pattern of S100A4 from different compartments would in principle argue against such hypothesis. However, even with apparent qualitative similarity in observed 2D patterns subtle quantitative differences, not apparent by western blot or silver staining analysis, might exist between corresponding spots from different sources, reflecting stochiometric variation of unmodified and modified S100A4. Recently, the modification of S100A8 and S100A9 by introduction of *N*^ε^-carboxymethyllysine was demonstrated to be associated with sustained intestinal inflammation through NFκB activation [42]. The addition of extracellular S100A4 has been shown to induce NFκB activity in a low S100A4 expressing osteosarcoma cell line [19], but the possibility of a PTM being involved in such activation has yet to be explored.

At present we do not know which PTMs are present in S100A4 or the location in the amino acid sequence, but speculations regarding the purpose of possible PTMs can be made. PTMs in the hinge region could affect steric properties, while changes to the C-terminal end, which is exposed upon calcium binding, could directly affect the proteins interacting capabilities. Both hinge and C-terminal regions have, based on their heterogeneity in the S100-family [8], been proposed to be important for the biological activity of each S100-protein [43], which further suggests that PTMs in these regions might also be specific to each S100-protein. One of the main features of the S100-proteins is their calcium binding motifs, the EF-hands, and which have also been shown to be important for dimerization and biological functions [6], is also the most conserved region among the S100 family members. PTMs introducing a negative charge or neutralizing an initially positive charge could potentially increase the calcium binding affinity [8], and the 2D pattern observed for S100A4 with gradually more acidic variants could thus suggest increased calcium binding capacity. For S100A9 and S100A12, phosphorylation of at least two different amino acids was shown to induce augmented calcium binding, which subsequently led to plasma membrane translocation of the proteins [44]. Since the calcium-binding domains are highly conserved, a general PTM that affects several of the S100-members could be possible. However, posttranslational modifications made directly to the EF-hands in the family of EF-hand proteins have to our knowledge not yet been described, and would therefore be of great interest in an even broader perspective.

## Conclusion

Endogenously expressed S100A4 was demonstrated to exist in several charge variants, indicating the presence of posttranslationally modified forms of the protein. These charge variants appeared to be universially present in S100A4 isolated from different subcellular compartments and tissues. As part of the emerging field of oncoproteomics, PTMs are proving to be increasingly relevant for the understanding of cancer biology and as potential drugable targets [45,46]. S100A4 has in numerous studies already been established as an important determinant of cancer metastasis, and potential PTMs may be of relevance for determining the biological processes resulting in increased metastatic capacity. Further efforts will be made to discover the nature of the PTMs present in S100A4 by optimizing mass spectrometry strategies and to further screen for eventual differential presence of the PTM using 2-D Fluorescence Difference Gel Electrophoresis (DIGE).

## Authors' contributions

MHH carried out the molecular work, mass spectrometric study and drafted the manuscript. KF conceived the study, participated in its design and coordination, and helped to draft the manuscript. S–OM participated in the design of the study and training MHH in mass spectrometry techniques. GMM conceived the study, participated in its design and coordination, and helped to draft the manuscript. All authors read and approved the final manuscript.

## Pre-publication history

The pre-publication history for this paper can be accessed here:


